# Field Trapping and Flight Capacity of *Eucosma giganteana* (Riley) (Lepidoptera: Tortricidae) in Response to Behaviorally Active Congeneric Semiochemicals in Novel Silflower Agroecosystems

**DOI:** 10.3390/insects13040350

**Published:** 2022-04-01

**Authors:** Kaitlyn P. Ruiz, Alexander Bruce, Nervah E. Chérémond, Chase A. Stratton, Ebony G. Murrell, Samantha Gillette, William R. Morrison

**Affiliations:** 1 Center for Grain and Animal Health Research, Agricultural Research Service, USDA, 1515 College Ave, Manhattan, KS 66502, USA; ruizkaitlyn97@gmail.com; 2 Department of Entomology and Plant Pathology, University of Tennessee, Knoxville, TN 37996, USA; alexbruce89@gmail.com; 3 The Land Institute, 2440 E. Water Well Rd., Salina, KS 67401, USA; cheremond@landinstitute.org (N.E.C.); stratton@landinstitute.org (C.A.S.); murrell@landinstitute.org (E.G.M.); 4 Department of Animal Sciences, Kansas State University, 2900 College Ave., Manhattan, KS 66502, USA; sagillette@ksu.edu

**Keywords:** flight behavior, attractants, integrated pest management, silflower, rosinweed, behaviorally-based management, (*E*)-8-dodecenyl acetate, (*Z*)-9-dodecenyl acetate

## Abstract

**Simple Summary:**

Rosinweed is a novel perennial crop being developed for oilseed and biofuel in the midwestern US. A primary pest is the Giant Eucosma Moth (GEM). Little is known about the chemical ecology or flight behavior of adults, but many attractants have been identified from other closely related species. The goals of this study were to evaluate whether any of these compounds could improve the capture of GEMs on sticky cards in the field and determine whether the most attractive volatiles might affect flight behavior in the laboratory. We found that there was significant attraction to (*E*)-8-dodecenyl acetate, which may possibly be a component in the pheromone blend for GEM. Exposure to these compounds in the laboratory reduced flight capacity. Our results suggest these two compounds could be included in monitoring or management programs for the Giant Eucosma moth.

**Abstract:**

*Silphium integrifolium* is a novel perennial crop being developed for oilseed and biofuel in the midwestern US. One of the primary pests in this system is *Eucosma giganteana* (Lepidoptera: Tortricidae). Little is known about the chemical ecology or flight behavior of *E. giganteana*, but many semiochemicals have been identified from other closely related *Eucosma* species. Some of these compounds include: (*Z*)- and (*E*)-8-dodecenyl acetate, (*E*)-9-dodecenyl acetate, (*Z*)-8-dodecenol, (*E*,*E*)-8,10-dodecadienyl acetate, and (*Z*,*E*)-9,12-tetradecadienyl acetate. The goals of this study were to evaluate whether any of these compounds could improve capture of *E. giganteana* on clear sticky cards in the field, and the most attractive volatiles might affect flight behavior on a computer-automated flight mill assay. We found that there was significant attraction to (*E*)-8-dodecenyl acetate in two years in the field, which may possibly be a component in the pheromone blend for *E. giganteana*. On flight mills, *E. giganteana* flew an average of 23 km in a 24 h period. The presence of attractive stimuli (e.g., (*E*)-8-dodecenyl acetate) had arresting properties and decreasing flight distance on the mill by 78 to 80%. The longest flight distances were registered in the morning (4:00–12:00) and were 1.8-fold greater than flight distances and durations at night (20:00–4:00). (*E*)-8-dodecenyl acetate may be useful in behaviorally based monitoring and management strategies for *E. giganteana*. Overall, our research expands the knowledge on the chemical ecology of adult *E. giganteana*.

## 1. Introduction

*Silphium integrifolium* Michx., or silflower, is a perennial plant that is native to the prairies of North America [1]. This novel crop is being developed for oilseed and biofuel needs in the midwestern US [2,3,4]. Silflower has an improved sustainability profile compared to canola due to its ability to survive short-term drought by accessing groundwater through extensive roots [5]. As rainfall patterns become increasingly unpredictable in the High Plains [6], this trait will become increasingly important. This plant also produces a thick resin when the integument is pierced by insects or mechanical damage, providing enhanced defenses compared to domesticated crops. There is an ongoing breeding program to domesticate *S. integrifolium* and enhance seed yield, so it can substitute for current annual oilseed crops (e.g., [1,3]).

One of the primary pests in this system is *Eucosma giganteana* (Riley) (Lepidoptera: Tortricidae), a specialist of *Silphium* spp. This species is thought to be univoltine, with adult flight taking place during early to mid-summer in Kansas, coinciding with the time that silflower blooms (Murrell, pers. comm.). Adults mate and lay eggs on inflorescences, then the larvae hatch and bore into the heads of the flowers. After feeding on the flower heads for several weeks, the larvae then descend and burrow into the root crowns, where they feed and weaken the plant’s ability to store carbohydrate reserves for future years [7]. Prior work found that the presence of *E. giganteana* reduced seed production in infested flower heads by 45–85% versus undamaged heads [8]. This species has been identified as one of the key limiting factors to the domestication of *S. integrifolium* in and around Kansas [8,9]. Furthermore, infestation by *E. giganteana* in North Dakota eliminated viable seeds in nursery plants of the closely related *S. perfoliatum* [10]. Given the economic damage and challenges posed by this moth on breeding efforts for *S. integrifolium*, effective pest management tactics are imperative.

A variety of approaches have been evaluated for the management of *E. giganteana*. Vilela et al. [9] unsuccessfully attempted to reduce *E. giganteana* colonization by trimming whole *S. integrifolium* plantings to shift their flowering date to later in the season; while the flowering date was shifted, this did not result in decreased pest infestations. A single application of 0.4% permethrin provided a three-fold reduction in *E. giganteana* infestation compared to untreated controls. Nonetheless, permethrin has broad spectrum activity against many taxa, including pollinators, and many pollinators use *S. integrifolium* as a resource [8]. Additionally, while *S. integrifolium* has some ability to self-pollinate, its seed yield benefits greatly from cross-pollination [11]. As a result, there is a need for more selective management strategies that spare pollinators.

One class of alternative IPM tactics are novel semiochemical-based monitoring and behaviorally based management approaches, whereby pest populations are manipulated at a distance with semiochemicals and removed from the foraging population with targeted insecticide use or by virtue of their biology [12]. Overall, there is a dearth of information on the basic behavioral and chemical ecology of *E. giganteana*. For example, the sex pheromone is unknown for this species, and no one has evaluated attractants, repellents, or other semiochemicals for this pest, either in the field or laboratory. However, a great deal more is known about related *Eucosma* species. For example, the pheromone of *Eucosma nothanthes* was isolated and discovered to be (*Z*)-8-dodecenyl acetate and (*Z*)-8-dodecenol [13]. Furthermore, in the tribes Eucosmini and Grapholitini, isomers (*E*,*E*)-, (*E*,*Z*)-, (*Z*,*E*)-, and (*Z*,*Z*)-8,10-dodecadien-l-yl acetate were classified as conserved sex pheromone components or attractants, including for *Eucosma* spp. [14]. A combination of (*E*)-9-dodecenyl acetate, (*Z*)-9-dodecenyl acetate, and (*E*,*E*)-8,10-dodecadienyl acetate captured *Eucosma bobana* Kearfott, *Eucosma ponderosa* Powell, and *Eucosma recissoriana* complex [15]. Furthermore, Frérot et al. [16] found that *Eucosma cana* Haw. and *Eucosma cumulana* Guin. were attracted to and trapped by lures with (*E*)-8-dodecenyl acetate, (*Z*)-8-dodecenyl acetate, and (*E*)-8-dodecen-1-ol in apple orchards. The structural commonality in attractants for related species suggests that they may also exhibit behaviorally relevant activity for *E. giganteana*.

Finally, there is very little known about the flight dynamics of *E. giganteana*, including its baseline flight capacity. In other Lepidopterans, flight capacity may be surprisingly high for small-sized species in the same family. For example, female flight capacity for female *Cydia pomonella* L. (Lepidoptera: Tortricidae) can reach up to 11 km [17]. Importantly, flight behavior may change in response to the presence of semiochemicals, as was demonstrated when the sex pheromone and food cues were present for *Ephestia kuehniella* Zeller (Lepidoptera: Pyralidae) [18]. Flight behavior may change over the diurnal time course; the total flying distance in 60 min varied with time of day for *Cactoblastis cactorum* (Berg) (Lepidoptera: Pyralidae), during dusk and the beginning of the scotophase [19]. Thus, the dispersal capacity of *E. giganteana*, adult diurnal activity patterns, and its flight responses to semiochemicals all remain open questions.

The goal in the current study was to evaluate attraction of *E. giganteana* to semiochemicals identified from congeneric *Eucosma* when paired with clear sticky traps, and determine how the most attractive ones affect flight capacity in the laboratory. These compounds may be useful in behaviorally based monitoring and management strategies. We hypothesized that attractive semiochemicals in the field would reduce flight capacity in the laboratory. Overall, we expect that the knowledge gained from this study would greatly expand our understanding of the behavioral and chemical ecology of *E. giganteana*.

## 2. Materials and Methods

### 2.1. Cropping System and Field Sites

All traps were deployed at three fields of actively managed *S. integrifolium* breeding plots in East-Central Kansas at The Land Institute (Table 1). Fields were located 0.6–2.5 km apart from each other and were 3–5 years old at the time this study was conducted. Inter-rows were planted with perennial grasses, which were mowed for weed management, but no pesticides were applied. The surrounding landscape consisted of a mix of hardwood species windbreaks, intermediate wheat grass cultivated for Kernza^®^ (The Land Institute, Salina, KS, USA), perennial sorghum, and blends of fescue.

### 2.2. Source Insects for Laboratory Bioassays

Field-captured *E. giganteana* adults were used for laboratory data collection because it is not currently possible to rear the species in the laboratory. Individuals were collected from a combination of two UV light traps deployed at The Land Institute in Salina, KS, USA, and hand-collected at night during the period of peak activity for *E. giganteana*. Individuals were collected on host silflower plants from 20:30–22:00 from multiple field sites between June and August in 2019 and 2020. Both sexes were equally represented from collections on flowers. Adults were held no more than 24 h at constant temperature (23 ± 0.1 °C) and 60 ± 3% RH in an environmental chamber before use in an assay. Adults were mixed ages but at least 24 h old.

### 2.3. Semiochemicals Treatments

Eight semiochemicals were identified as attractants from other related *Eucosma* spp. through the primary literature [13,14,15,16,20,21,22,23,24] and searching Pherobase [25]. Semiochemicals were purchased from ALFA Chemistry (Ronkonkoma, NY, USA), TRC Chemistry (North York, ON, Canada), and Bedoukian Inc. (Danbury, CT, USA), and formulated in behaviorally-relevant concentrations emitted by other *Eucosma* spp. (Table 2). Each of the eight semiochemicals was serially diluted with acetone and 2.5 mL of each was pipetted in 3-mL LDPE dropping bottles (Wheaton, DWK Life Sciences, LLC, Millville, NJ, USA). Concentrations varied from 0.004 to 0.02 µg/μL, depending on the behaviorally relevant dose (see above). Each was compared to a negative control consisting of 2.5 mL of acetone (solvent only).

### 2.4. Field Trapping Assay

Each field had three transects spaced at least 10 m apart, each with a full set of semiochemical treatments represented. Each trap within the transect was spaced 10 m apart. Each trap consisted of a 1.27-cm diameter PVC pipe hammered in row with the silflower to a finished height of 1 m, in line with the canopy of *S. integrifolium*. A single 30.4 cm × 30.4 cm clear sticky card (Alpha Scents, Canby, OR, USA) was folded in half and inserted in a 271 cm long sticky card ring holder (Olson Products Inc., Medina, OH, USA). The ring holder was bent at a 90° angle to wedge the card holder upright in position, which was subsequently wedged in the opening at the top of the PVC pipe. To protect against dislodgement by wind, sticky cards were affixed to the top of the metal card holder with a 50 mm × 30 mm binder clip. A single, capped LDPE 3-mL dropping bottle with one of the semiochemical treatments above was inserted in the top of the PCV pipe opening and affixed in place by tying it to the card holder with garden wire. Every week, the lures were replaced with a freshly prepared treatment and the position of the lure was rotated in the transect every two weeks. Traps were rotated because of the short duration of the flying season and resulted in every treatment occupying every position at least once. Sticky cards were changed on a weekly basis after the first recorded capture of an *E. giganteana* adult. Traps were deployed 7 June 2019 to 14 August 2019 and 15 June 2020 to 10 August 2020. In total, there were *n* = 3 replicates of each semiochemical treatment per field site. The number of *E. giganteana* and Lepidopteran nontargets was counted on each sticky card after freezing cards at −20 °C for at least 24 h.

### 2.5. Volatile Release Experiment

To confirm lure release rate over a two-week period, a volatile release rate experiment was performed, as has been conducted prior [26]. Each of the 8 semiochemical lures was aged 1, 3, 5, 7, and 14 d in a chamber with a constant temperature of 31.7 °C and a RH of 68%. These were comparable to the average temperature and RH in Salina, KS, USA, at The Land Institute from June to August during the field trapping assay. Central air was guided through an activated charcoal, which ran through PTFE inert tubing to flow meters at 1 L/min and into glass, nonporous 500-mL capacity headspace chambers, containing capped lures (e.g., LDPE dropping bottles with semiochemicals). Headspace was collected on volatile collection traps, which consisted of 7.62-cm-long tapered borosilicate glass tubes (0.64 cm diameter) packed with 20 mg of Porapaq-Q adsorbent material and sandwiched with a PTFE-retaining plug and borosilicate wool on one side and a mesh wire screen (SS-316) on the other. Afterwards, volatiles were eluted with 150 µL of dichloromethane into a 2-mL GC vial with a glass insert containing polymer feet. A total of 1 μL of a tetradecane internal standard (190.5 ng) was added to each sample, then analyzed using gas chromatography coupled with mass spectrometry as below. There were *n* = 3 replicates per treatment and period combination.

### 2.6. Gas Chromatography Coupled with Mass Spectrometry

All headspace collection sample extracts were run on an Agilent 7890B gas chromatograph (GC) equipped with an Agilent Durabond HP-5 column (30 m length, 0.250 mm diameter and 0.25 μm film thickness; Santa Clara, CA, USA) with He as the carrier gas at a constant 1.2 mL/min flow and 40 cm/s velocity, which was coupled with a single-quadrupole Agilent 5997B mass spectrometer (MS). The compounds were separated by auto-injecting 1 µL of each sample under split mode into the GC-MS at room temperature (approximately 25 °C). The flow was split in a 15:1 ratio with a split flow rate of 18 mL/min. The program consisted of 40 °C for 1 min followed by 10 °C/min increases to 300 °C and then held for 26.5 min. After a solvent delay of 3 min, mass ranges between 50 and 550 atomic mass units were scanned. Compounds were tentatively identified by comparison of their spectral data with those from the NIST 17 library and by GC retention index. Using the ratio of the peak area for the internal standard to the peak area for the other compounds in the headspace, the emission rates of samples in ng of volatile per μL of solvent and per h of collection were calculated. Identity of semiochemical lures and location on chromatogram was confirmed by injecting a diluted sample of a stock solution of pure compound.

### 2.7. Flight Mill Apparatus

Six flight mills were situated on a 71.1 cm × 91.4 cm sheet of nonporous plastic to minimize vibrations. Each mill was spaced 39 cm apart in rows (e.g., 3 in a row), and 25.4 cm between rows. Each mill contained three wires connected to the main connector board (#777101-01, National Instruments, Austin, TX, USA). A DC Power Supply (QW-MS3010D, Wuxi Qiaowei Eelectroeics Co., Ltd., Wuxi, Jiangsu, China) was set to the site and connected to the main wiring block to provide power. A 50-pin ribbon cable (180524-10, National Instruments) ran from the main wiring connector block into a specially made PC port (77690-01, National Instruments), where a computer with the software Labview 2017 (version 17.0.1f3, National Instruments) automatically recorded the data from the mills. Each mill consisted of a PTFE cylinder with a head suspended by two antipodal magnets to create levitation and connected by an insect pin with lubricant to ensure frictionless turning. At the top of the head, 2 14 cm hollow tubes (total flight diameter was 28 cm and circumference was 0.86 m) extended perpendicularly with right angles formed at the end for insect attachment. Protruding from the bottom of the head was a magnet to activate the Halls Sensor placed on the base of the mill during rotation. Levels on the bottom of the mills and adjustable feet ensured that mills were even relative to center of gravity. Lighting was uniformly provided overhead using full spectrum lights.

### 2.8. Flight Mill Assay

Six adults were run simultaneously on the six flight mills described above (15-FMASM SDP Unit, Crist Instrument Co., Hagerstown, MD, USA) to test flight capacity. A 14-gauge copper wire was stripped into individual threads and cut into 4 cm segments. The copper wire thread was wrapped around insect pin (#6 insect pin, BioQuip Products, Rancho Dominguez, CA, USA) embedded in modelling clay to form a loop. The loop was pinched with a standard metal forceps, then excess wire was ablated on the shorter end with scissors, and the loop was flattened relative to the plane of gravity. Moths were placed singly on a metal mason jar lid on top of ice and restrained with a plastic lid for a 59.1-mL container until sessile. Afterwards, the pronotum of *E. giganteana* was descaled lightly with an artist’s paintbrush; then the copper loop was dipped in instant adhesive (#347908, Evo Stik Multi-Purpose Impact Adhesive, Bostik, Ltd., Leicester, UK) and affixed onto the pronotum of the adult, ensuring not to impair proper wing functioning, and avoiding the eyes and antennae of the adults. The individual was tethered by inserting the point of the copper wire of the hypodermic needle attached to the rotation arm of each flight mill. Each trial was started between 15:00 and 18:00 by gently blowing on the insect to initiate flight and run for 24 h in parallel. Assays were conducted at 21.4 ± 0.01 °C temperature and 54.2 ± 0.2% RH and monitored with a datalogger (UX100-011, Hobo, temp/RH logger, Onset, Bourne, MA, USA). The semiochemical treatments in the flight mill assay included (*E*)-8-dodecenyl acetate, (*Z*)-9-dodecenyl acetate, and an unbaited control (acetone solvent only). Semiochemicals were freshly prepared in LDPE dropping bottles, as in the field-baiting experiment (as above), and placed in the center among the flight mills on a nonporous glass surface to prevent contamination. A smoke test confirmed that volatiles came into contact with each flight mill. Between runs, surfaces on the flight mill were thoroughly wiped down with solvent and allowed to dry and dissipate before use again. There were 12–18 replicates per treatment. At the end of a trial, insects were detached and weighed on a balance. Data were streamed in real time to a computer which automatically record the flight parameters: distance flown, the number of tandem flight bouts over the sampling interval (flights lasting more than 1 s), mean flight bout duration, and mean distance flown per bout. Data were also parsed by diurnal period (morning, afternoon, and night) to determine maximum time of dispersal. Periods were defined as follows: Morning: 4:00–11:59; Afternoon: 12:00–19:59; Night: 20:00–3:59. Data were analyzed with R software.

### 2.9. Statistical Analysis

The trapping data were analyzed with a separate repeated measures linear mixed model for each year that used either *E. giganteana* or the number of Lepidopteran nontargets as the response variable. A first order autoregressive variance–covariance matrix was used to account for autocorrelation among sampling dates. Date was treated as a random variable. The fixed explanatory variable included semiochemical treatment (including those treatments listed in Table 2). Residuals were inspected to ensure assumptions of normality and homogeneity of variance was fulfilled. Where there were deviations from assumptions, data were log-transformed, after which assumptions were fulfilled. Upon a significant result from the model, Tukey HSD was used for multiple comparisons. R Software (R Core Team 2020) was used for all analyses, with α = 0.05, unless otherwise specified.

A multivariate analysis of variance (MANOVA) was used to analyze the data from the flight mill. In particular, the total distance flown in 24 h (km), number of flight bouts, duration of flight bouts (s), and distance flown in each flight bout (m) was coded as an aggregate response variable. Semiochemical treatment (e.g., Table 2) was used as a fixed, explanatory variable. Deviations from normality or homogeneity of variances were corrected via log-transformations. Upon a significant result from the MANOVA, sequential ANOVAs were performed on each response variable. Upon significant result from the ANOVA, Tukey HSD was used for multiple comparisons.

## 3. Results

### 3.1. Field Trapping Assay

**Figure 1:**
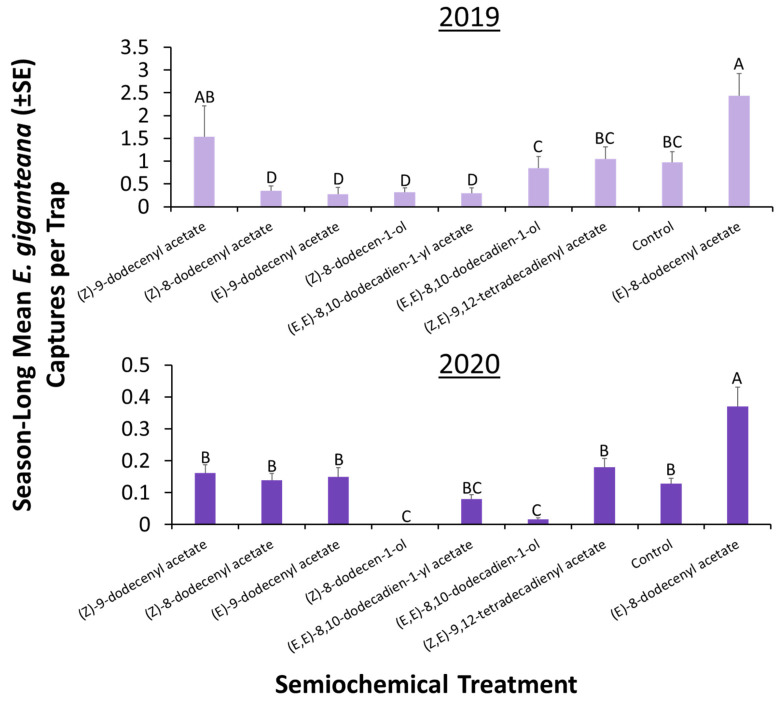
Season-long mean weekly trap capture (±SE) of *E. giganteana* on clear sticky cards deployed at 1-m height in row with *Silphium integrifolium* and baited with congeneric semiochemicals from related *Eucosma* spp. in Salina, KS, USA, at the Land Institute in from June to August 2019 (top panel) and 2020 (bottom panel). There was a total of *n* = 3 replicate fields used with 3 sets of replicate treatments at each field. Bars with shared letters are not significantly different from each other (Tukey HSD, α = 0.05). In both years, (*E*)-8-dodecenyl acetate was a significant attractant to *E. giganteana*.

**Figure 2:**
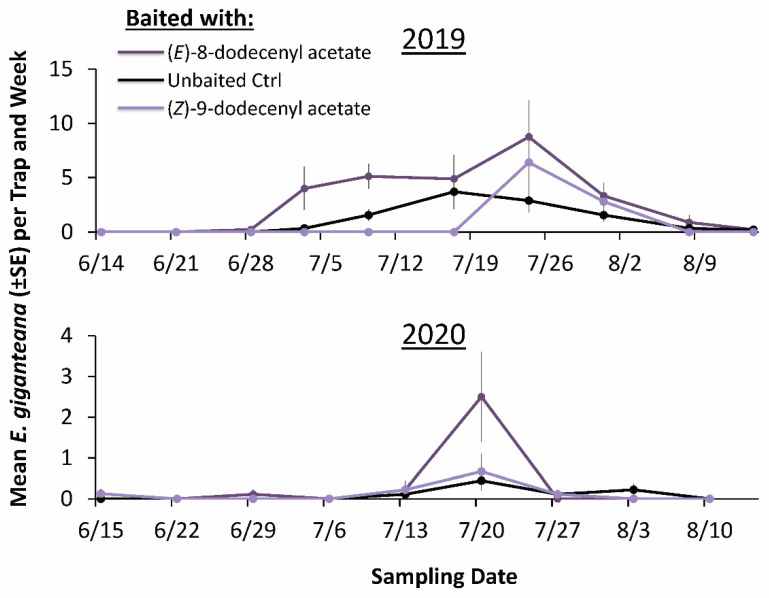
Population dynamics of *E. giganteana* trap capture (±SE) over the course of the season on clear sticky cards deployed at 1-m height in row with *Silphium integrifolium* and baited with the congeneric semiochemicals capturing the highest number of moths (e.g., (*E*)-8-dodecenyl acetate, dark purple line) and lowest number of moths (e.g., (*Z*)-9-dodecenyl acetate, light purple line) compared to the unbaited control (black line) in Salina, KS, USA, at the Land Institute in from June to August 2019 (top panel) and 2020 (bottom panel). There was a total of *n* = 3 replicate fields used with 3 sets of replicate treatments at each field.

In total, 651 *E. giganteana* moths were captured on sticky cards in 2019. The semiochemical lures significantly affected *E. giganteana* moth capture (Repeated Measures LMM: χ^2^ = 54.7; df = 8; *p* < 0.0001), with 2.5-fold more captures on sticky cards with (*E*)-8-dodecenyl acetate compared to the unbaited control (Figure 1). In fact, over the course of the season, sticky cards baited with (*E*)-8-dodecenyl acetate captured 36% of the total moths during the season (Figure 2). Next, (*Z*)-9-dodecenyl acetate was the second most attractive compound to *E. giganteana*, capturing 1.6-fold more moths when paired with clear sticky traps than the unbaited control (Figure 1). By contrast, (*Z*)-8-dodecenyl acetate, (*E*)-9-dodecenyl acetate, (*Z*)-8-dodecen-1-ol, and (*E*,*E*)-8,10-dodecadien-1-yl acetate captured 28–35% of the moths found on the unbaited control sticky cards, suggesting repellency or active inhibition (Figure 1).

In 2019, 3225 nontarget Lepidoptera were captured on the clear sticky traps. However, the semiochemical lure paired with sticky cards did not significantly affect the number of nontarget Lepidoptera (Repeated Measures LMM: χ^2^ = 14.4; df = 8; *p* = 0.07). Each semiochemical treatment captured 6–14% of the total nontargets on the sticky cards over the course of the season, with a mean number of 4–9 nontarget Lepidoptera per card (Table 3).

In 2020, a total of 76 *E. giganteana* moths were collectively captured on sticky cards. The semiochemical lures significantly affected *E. giganteana* capture (Repeated Measures LMM: χ^2^ = 17.1; df = 8; *p* < 0.01). Like the prior year, there were 2.9-fold more captures of moths on sticky cards with (*E*)-8-dodecenyl acetate compared to the unbaited control (Figure 1). Moreover, sticky cards baited with (*E*)-8-dodecenyl acetate captured a third of the total moths during the season (Figure 2). Similarly to 2019, traps baited with (*Z*)-8-dodecen-1-ol captured the fewest moths among the treatments, with zero captures the entire season, while (*E*,*E*)-8,10-dodecadien-1-ol reduced captures on sticky cards by 88% compared to unbaited controls, suggesting active inhibition.

In 2020, 2164 nontarget Lepidoptera were captured on the clear sticky traps. However, the semiochemical lure paired with sticky cards did not significantly affect the number of nontarget Lepidoptera (Repeated Measures LMM: χ^2^ = 5.86; df = 8; *p* = 0.66). Each semiochemical treatment captured 10–14% of the total nontargets on the sticky cards over the course of the season, with a mean number of 3–5 nontarget Lepidoptera per card (Table 3).

### 3.2. Volatile Release Experiment

**Figure 3:**
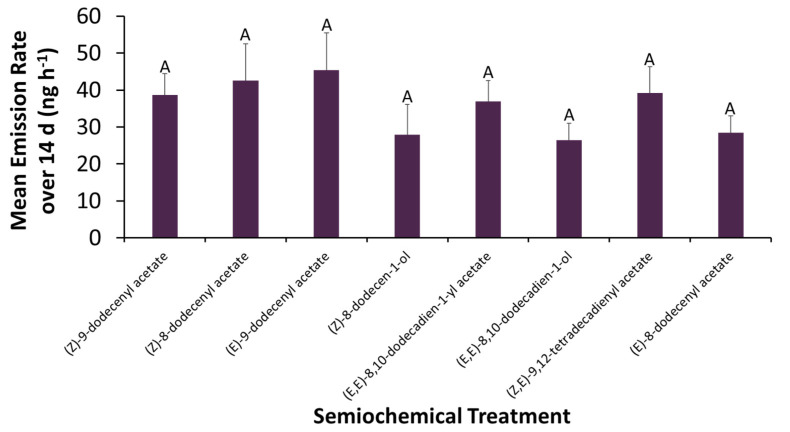
Mean volatile emission rates (±SE) by semiochemical lures used in the field baiting experiment after aging over average Salina, KS, USA, summer conditions in an environmental chamber (31.7 °C, 68% RH) for a 14-d period and sampling at 1, 3, 5, 7, and 14 days with *n* = 3 replicates per compound and time point. Bars with shared letters are not significantly different from each other (Tukey HSD, α = 0.05). All lures reliably emitted semiochemicals over their period of use in the field in statistically similar amounts to each other.

The mean emission rates among the lures used in the field baiting experiment were statistically similar over a 14-d period (Repeated Measures ANOVA: *F* = 1.83; df = 7, 15; *p* = 0.09), ranging between 27 ± 9.7 and 45 ± 10 ng·h^−1^ (Figure 3). The lure consisting of (*E*)-9-dodecenyl acetate and (*E*, *E*)-8,10-dodecadien-1-ol were numerically the highest and lowest emitter, respectively. Emissions lasted over the full sampling period.

### 3.3. Flight Mill Assay—24 h Period Analysis

**Figure 4:**
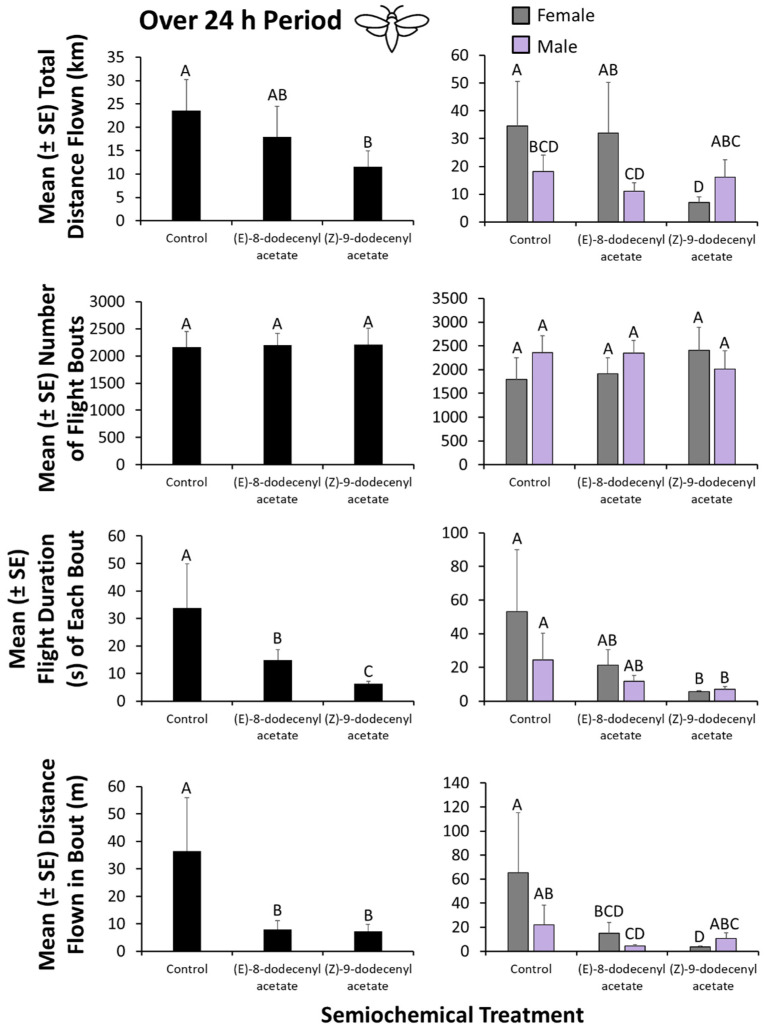
Characterization of flight behavior of *E. giganteana* on computer-automated flight mills in the laboratory over a 24-h period to evaluate the individual and combined effect of the presence of (*E*)-8-dodecenyl acetate and (*Z*)-9-dodecenyl acetate from the field baiting experiment above, as well as the sex (female—grey bars, male—purple bars) of an individual on flight capacity. A total of *n* = 18 replicate moths were tested per semiochemical treatment. Bars with shared letters are not significantly different from each other (Tukey HSD, α = 0.05).

Overall, the semiochemical present significantly affected the flight behavior of *E. giganteana* in the laboratory (MANOVA: Roy’s Approx. *F* = 17.4; df = 3, 34; *p* < 0.0001; Figure 4). Flight behavior also significantly varied according to the sex of the moth (Roy’s Approx. *F* = 3.80; df = 2, 34; *p* < 0.01) and the interaction between sex and semiochemical (Roy’s Approx. *F* = 3.15; df = 3, 34; *p* < 0.05). As a result, sequential ANOVAs were performed for each response variable.

The semiochemical present significantly affected the total distance flown by *E. giganteana* over 24 h (ANOVA: *F* = 3.84; df = 2, 36; *p* < 0.05). For example, the distance flown by *E. giganteana* was only approximately half as much in the presence of (*Z*)-9-dodecenyl acetate compared with no stimuli (Figure 4). The sex of moths also significantly affected total distance flown (*F* = 10.8; df = 1, 36; *p* < 0.01), with females flying 1.5-fold farther on average (Figure 4). The interaction between sex and semiochemical treatment was not significant (*F* = 1.31; df = 2, 36; *p* = 0.28). By contrast, neither the semiochemical treatment (*F* = 3.01; df = 2, 36; *p* = 0.06), sex (*F* = 0.14; df = 2, 36; *p* = 0.70), nor their interaction (*F* = 0.008; df = 2, 36; *p* = 0.99) affected the total number of flight bouts by *E. giganteana* (Figure 4).

The semiochemical present significantly affected the mean duration of each flight bout by *E. giganteana* on the flight mill (*F* = 9.96; df = 2, 36; *p* < 0.001). For instance, in the presence of (*Z*)-9-dodecenyl acetate and (*E*)-8-dodecenyl acetate, moths only flew 18% and 44% of the duration that control moths flew when no stimuli were present (Figure 4). However, sex did not affect the duration of each flight bout (*F* = 1.74; df = 1, 36; *p* = 0.19), nor did its interaction with semiochemical (*F* = 0.39; df = 2, 36; *p* = 0.68).

Furthermore, the semiochemical present significantly affected the distance flown in each flight bout (*F* = 30.6; df = 2, 36; *p* < 0.0001), with only 19–22% of the distance flown when (Z)-9-dodecenyl acetate and (*E*)-8-dodecenyl acetate were present compared to the unbaited control (Figure 4). The sex of the moth also significantly affected the distance flown in each flight bout (*F* = 12.9; df = 1, 36; *p* < 0.0001), with female moths flying 2.1-fold farther than males on average. The interaction between sex and semiochemical treatment significantly affected the distance flown in each bout (*F* = 4.13; df = 2, 36; *p* < 0.05). For example, distance flown in each bout by males was marginally reduced in the presence of (*E*)-8-dodecenyl acetate, while, for females, it was more reduced by (*Z*)-9-dodecenyl acetate compared to control moths.

### 3.4. Flight Mill Assay—8-h Diurnal Period Analysis

**Figure 5:**
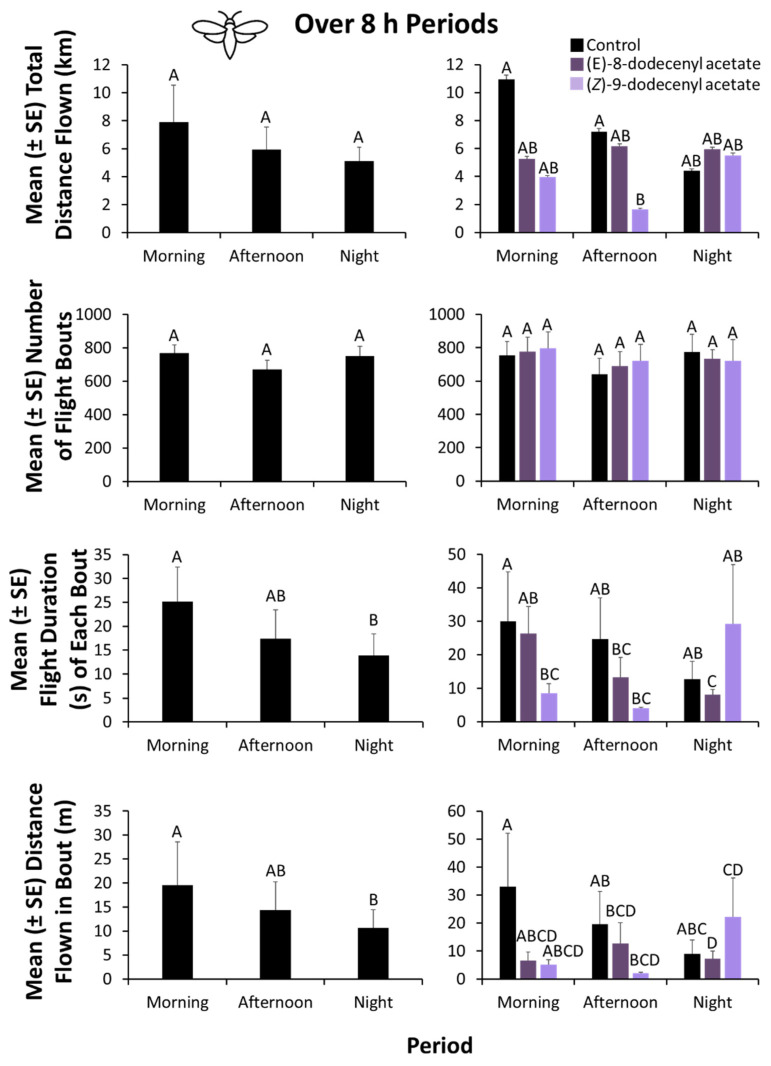
Characterization of flight behavior of *E. giganteana* on computer-automated flight mills in the laboratory at 8-h increments to determine optimum period of flight (morning, afternoon, or night) over the diurnal time course in the presence of (*E*)-8-dodecenyl acetate and (*Z*)-9-dodecenyl acetate from the field baiting experiment. A total of *n* = 18 replicate moths were tested per semiochemical treatment. Periods are defined as follows: Morning: 4:00–11:59; Afternoon: 12:00–19:59; Night: 20:00–3:59. Bars with shared letters are not significantly different from each other (Tukey HSD, α = 0.05).

Overall, the period significantly affected the flight behavior of *E. giganteana* in the laboratory (MANOVA: Roy’s Approx. *F* = 8.79; df = 2, 99; *p* < 0.0001; Figure 5). Moreover, the semiochemical lure (Roy’s Approx. *F* = 17.4; df = 2, 99; *p* < 0.0001), and its interaction with the period (Roy’s Approx. *F* = 4.76; df = 4, 99; *p* < 0.01) significantly affected the flight behavior of *E. giganteana*. As a result, sequential ANOVAs were performed for each response variable.

Whereas the period did not significantly affect the distance flown (ANOVA: *F* = 1.44; df = 2, 99; *p* = 0.24) by *E. giganteana*, the semiochemical lure did (*F* = 6.41; df = 2, 99; *p* < 0.01; Figure 5), but the interaction between the two did not (*F* = 1.18; df = 4, 99; *p* = 0.32). By contrast, neither the period (*F* = 0.013; df = 2, 99; *p* = 0.98), semiochemical lure (*F* = 2.79; df = 2, 99; *p* = 0.07), nor their interaction (*F* = 0.67; df = 4, 99; *p* = 0.61) affected the total number of flight bouts by *E. giganteana* (Figure 4).

The flight duration of each bout was significantly affected by period (*F* = 15.6; df = 2, 99; *p* < 0.0001), with *E. giganteana* moths flying 1.8-fold longer in the morning than in the night (Figure 5). The semiochemical lure also affected the flight duration in each bout by moths (*F* = 9.31; df = 2, 99; *p* < 0.001), but its interaction with period was not significant (*F* = 2.20; df = 4, 99; *p* = 0.07).

Similarly, the total distance flown by *E. giganteana* in each bout was significantly affected by period (*F* = 8.86; df = 2, 99; *p* < 0.001), with moths flying 1.8-fold farther in the morning than in the night (Figure 5). In addition, the semiochemical lure significantly affected the distance flown in each bout (*F* = 22.6; df = 2, 99; *p* < 0.0001), but its interaction with period was not significant (*F* = 0.31; df = 4, 99; *p* = 0.88).

## 4. Discussion

This is the first study to investigate behaviorally relevant semiochemicals and the flight behavior of *E. giganteana*. In both years of the field study, (*E*)-8-dodecenyl acetate, and to a lesser extent, (*Z*)-9-dodecenyl acetate, acted as an attractant when paired with a clear sticky card in the field for *E. giganteana*. The most attractive stimulus reliably captured moths throughout their period of flight each year. Both compounds and other structurally related chemicals have shown biological activity for related *Eucosma* species in prior studies. For example, *Rhyacionia zozana* (Kearfott) (Lepidoptera: Olethreutidae) was attracted to the closely related (*E*)-9-dodecenyl acetate, whereas *Eucosma sonomana* was attracted to a mix of the *Z*- and *E*- isomers of this compound [23]. In fact, a 4:1 mixture of (Z)-9 and (*E*)-9-dodecenyl acetate when used in a mating disruption strategy successfully suppressed damage by *E. sonomana* in 19 ha of forest, reducing damage by 67 to 79% [27]. The sex pheromone of *Eucosma coniogramma* was described as (*E*)-9-dodecenyl acetate as the major component, which was attractive to conspecifics in the field and produced a robust GC-EAD response [28]. However, the addition of (*E*)-8-dodecenyl acetate to (*Z*)-8-dodecenyl acetate on traps significantly decreased trap captures of *Eucosma notanthes* [21]. Rather, (*Z*)-8-dodecenyl acetate and (*Z*)-8-dodecenol have been described as the two-component sex pheromone for *E. notanthes* [13]. In pine plantations, *Eucosma monitorana* Heinrich, *Eucosma gloriola* Heinrich, and *Eucosma tocullionana* Heinrich were attracted to lures containing (*Z*)-9- and (*E*)-9-dodecenyl acetate in various ratios with or without (*Z*)-9-dodecen-1-ol [29]. In the field, *Eucosma womonana* Kearfott was captured by traps baited with a blend of five acetates, including (*Z*)-5-dodecenyl acetate and (*Z*)-7-dodecenyl acetate [30]. Notably, the sex pheromone of the oriental fruit moth, *Grapholita molesta* (Busck) (Lepidoptera: Tortricidae), has been described as a combination of (*Z*/*E*)-8-dodecenyl acetates [22], and a blend of these isomers in the ratio of 93:6, when also combined with (*Z*)-8-dodecen-1-ol, has proven highly effective when implemented using SPLAT technology for mating disruption of the species in apple orchards [24]. Overall, 12-carbon acetates appear important for the chemical ecology of *Eucosma* spp., which is why 5 were included among the semiochemical treatments in this study. Our study strongly suggests that (*E*)-8-dodecenyl acetate is a component of *E. giganteana* pheromone blend, although it is likely a minor one given a small 3-fold increase in attraction over control. Furthermore, it attracted the highest number of *E. giganteana* moths in both years and consistently over the course of the growing season. Future work should determine the other components of the pheromone blend and evaluate whether it can be used for mating disruption.

According to trap catches with (*E*)-8-dodecenyl acetate, the peak periods of flight of *E. giganteana* were 28 June to 9 August in 2019 and 15 June to 3 August 2020. While other work has monitored larval development through the season for *E. giganteana* [9,10], season-long trapping of adults has rarely been performed. Seasonal flights of *E. gloriola*, *E. monitorana*, and *E. tocullionana* were from 8 May to 4 June, 15 May to 26 June, and 22 May to 26 June 1991 in Ontario, Canada [29]. The endangered *Eucosma scorzonerana* was shown to have a flight period from the end of May to the middle of June in Sweden [31]. By comparison, flight starts later for *E. giganteana* and lasts longer than for other *Eucosma* species at higher latitudes.

We found that without stimuli, *E. giganteana* fly an average of 23 km in a 24 h period. This suggests that *E. giganteana* moths are possibly capable of being a landscape-level risk and may possibly be able to emigrate large distances to find new host patches if conditions are suboptimal in their current location. Under field conditions, suboptimal conditions may be less likely to be present, with an abundance of food and mates probably deterring long distance flights. Furthermore, as other authors such as Miller et al. [32] have noted, most insects are random walkers, and if *E. giganteana* falls into this group, then the total distance flown may not directly correlate with dispersal ability. Furthermore, it is notable that the mean distance flown in each flight bout is 36 m, which typically lasts only 30 s. The implication here is that while the total distance flown over an extended period can be large, it likely accumulates in small bouts of flight with individuals flitting from plant to plant in a field or among adjacent fields. This corresponds with field observations of *E. giganteana* moths flying from plant to plant or from the ground vegetation to plant during nocturnal collection periods (Morrison, pers. obs.). To assess whether *E. giganteana* is a random walker, release–recapture studies should be performed to see the maximum distance that they can fly from release points around a trap. Males appeared to be preferentially arrested by the presence of (*E*)-8-dodecenyl acetate, compared to females, which were more arrested in flight behavior by (*Z*)-9-dodecenyl acetate. A potential explanation for this may be because the former compound more closely resembles the natural sex pheromone, while the latter may indicate competing species and availability of preferred oviposition sites. Thus, for separate proximal reasons, both males and females exhibit shorter flights and arrestment when exposed to these compounds. There have been no prior studies on the flight behavior of *E. giganteana*, although *E. monitorana* was described as a weak flyer [33]. By contrast, *E. giganteana* appears to be a strong flyer, capable of extended, directed flight (Supplemental Video S1).

The presence of attractive stimuli reduced the distance flown in each flight bout by an average of 78 to 80%. This suggests that both (*E*)-8-dodecenyl acetate and (*Z*)-9-dodecenyl acetate had an arresting function on the behavior of *E. giganteana*. Both compounds appear to affect the flight behavior of *E. giganteana*. Interestingly, (*Z*)-9-dodecenyl acetate was just as arresting, even though it was not nearly as attractive in the field baiting experiment. This raises the possibility that blends of the semiochemicals used in this study may have synergistic properties. In a meta-analysis, Szendrei and Rodriguez-Saona [34] found that lures with an increasing number of compounds in the blends often outperformed lures with individual components. In the future, lures with combinations of 12-carbon acetates should be tested for their response by *E. giganteana* to determine if there is a synergistic response.

Although the total distance flown was not affected by the flight period, the longest flight bouts of *E. giganteana* in both duration and distance occurred in the morning (4:00–12:00). Adult *E. giganteana* flew 1.8-fold farther in each flight bout and 1.8-fold longer in the morning than at night. In other related species, adult *Eucosma obumbratana* are primarily active at sunset [35,36], whereas *E. scorzonerana* are not only active at sunset but also in early morning and late afternoon [37]. Night-time flights were significantly shorter in each bout, possibly due to mate-seeking behavior, which may require many flights of shorter duration and flight distance. Given the fact that there is a limited window in which to find a mate and the short adult lifespan of *E. giganteana*, it may be reasonable to expect that mate-finding would drive flight behavior during the period of peak activity.

We have included tests of both male and female response to potential pheromones in this study. Historically, female moths and their response to pheromones have been understudied component of the literature compared to their male counterparts. Prior work has shown that female autodetection of pheromone occurs in two important agricultural tortricids, affecting their flight behavior and having important implications in mating disruption programs [38]. One potential hypothesis is that female moth autodetection is in response to density in order to achieve optimal spacing. In the field, *E. giganteana* occurs at high densities, with multiple individuals per plant often widely spaced, making possible pheromone autodetection in this species. However, patches of silflower may be limiting, and thus the autodetection of pheromone by females may result in arrestment instead of flight because it could be an indicator of a feeding patch with other mating and oviposition opportunities that are relatively rare in the environment. As a result, both males and females were included in the study.

Among the other volatiles in this study, (*Z*)-8-dodecen-1-ol was also behaviorally active and consistently acted as a repellent to captures of *E. giganteana* on sticky cards. This information may be helpful, once the pheromone is identified, to know what trace impurities may hinder attraction to synthetic lures. In addition, depending on the behavioral dynamics displayed by *E. giganteana* in the presence of this compound, it may serve as a suitable semiochemical for incorporation in a push–pull strategy [12], possibly to protect silflower production on the Great Plains.

## 5. Conclusions

To our knowledge, this study provides new information about the chemical ecology, flight behavior, and dispersal capacity of *E. giganteana*. Future work should (1) collect headspace from *E. giganteana* conspecifics and perform glandular microdissections to identify the true sex pheromone, (2) confirm antennal response by *E. giganteana* to the compounds cited in this study by GC-EAD, and (3) evaluate combinations of 12-carbon acetates for synergistic response by moths. Furthermore, future work should relate trap captures of *E. giganteana* to *S. integrifolium* crop damage to develop pest management action threshold guidelines. This would be an additional tool in the IPM toolkit against *E. giganteana*. While this work has focused on testing congeneric pheromonal stimuli for behavioral response by *E. giganteana*, it is likely that host plant volatiles from *S. integrifolium* are also important for attraction by conspecifics. However, little is known about the headspace volatile profiles of silflower, or how it may influence the behavior of *E. giganteana,* so future work ought to include a rigorous evaluation of host plant volatiles. Ultimately, we have demonstrated that multiple volatiles may be useful in manipulating the flight behavior of *E. giganteana* and, when paired with clear sticky traps, provide a new IPM tool for monitoring *E. giganteana* populations.

## Figures and Tables

**Table 1:** Summary of field site characteristics for the trapping study in Kansas during 2019 and 2020.

Field ID	County	Latitude	Longitude	Area (ha)	Row Spacing (m)	Sprayed with Insecticide?	No. ^1^ of Transects w/Full Set of Trts
A1	Saline	38.770789	−97.5920072	0.381	2.2	No	2
A2	Saline	38.77233	97.5905690	0.160	1.8	No	1
B	Saline	38.76969	−97.5971841	0.939	3.3	No	3
C	Saline	38.770459	−97.5680977	0.340	1.0	No	3

^1^ Abbreviations: No.—number.

**Table 2:** Summary of semiochemical treatments and their concentrations in each lure for the field trapping study of *E. giganteana* at the Land Institute in Salina, KS, USA, in 2019 and 2020.

ID	Semiochemical Treatment	Purity	Starting Concentration from Literature	Concentration of Stock Solution	Volume (µL) of Stock Added to 2.5 mL of Acetone Lure	Final Amount of Each Cmpd in Lure	Manufacturer
1	(*E*)-8-dodecenyl acetate	95%	870 μg/μL	1.0 μg/μL	50	50 μg	ALFA Chem
2	(*E*,*E*)-8,10-dodecadien-1-ol	95%	10 μg/μL	10 μg/μL	1	10 μg	ALFA Chem
3	(*Z*)-8-dodecenyl acetate	95%	910 μg/μL	0.91 μg/μL	50	45.5 μg	ALFA Chem
4	(*Z*,*E*)-9,12-tetradecadienyl acetate	90%	1 μg/μL	1.0 μg/μL	50	50 μg	ALFA Chem
5	(*Z*)-8-dodecen-1-ol	95%	588.2 μg/μL	0.99 μg/μL	10	10 μg	TRC
6	Control (acetone)	99%	0 μg/μL	-	-	-	MilliporeSigma
7	(*E*,*E*)-8,10- dodecadien-1-yl acetate	95%	0.81 μg/μL	0.81 μg/μL	12.3	9.96 μg	Bedoukian
8	(*E*)-9-dodecenyl acetate	95%	0.81 μg/μL	0.81 μg/μL	12.3	9.96 μg	Bedoukian
9	(*Z*)-9-dodecenyl acetate	95%	0.81 μg/μL	0.81 μg/μL	12.3	9.96 μg	Bedoukian

**Table 3:** Mean (SE) nontarget lepidopteran captures on clear sticky cards changed on a weekly basis during the *E. giganteana* period of flight in two years from June to August 2019–2021 at the Land Institute in Salina, KS, USA.

Semiochemical Treatment	Nontarget Lepidopteran Captures per Week and Sticky Card			
	2019		2020	
	N ^1^	Mean ± SE	N	Mean ± SE
(*Z*)-9-dodecenyl acetate	30	6.0 ± 1.4 a ^2^	63	3.4 ± 0.2 a
(*Z*)-8-dodecenyl acetate	98	4.0 ± 0.6 a	65	3.6 ± 0.1 a
(*E*)-9-dodecenyl acetate	30	8.8 ± 1.6 a	61	3.9 ± 0.2 a
(*Z*)-8-dodecen-1-ol	99	3.8 ± 0.6 a	63	4.7 ± 0.2 a
*(E*,*E*)-8,10-dodecadien-1-yl acetate	30	6.6 ± 1.4 a	63	4.4 ± 0.2 a
(*E*,*E*)-8,10-dodecadien-1-ol	101	4.7 ± 0.7 a	63	3.8 ± 0.2 a
(*Z*,*E*)-9,12-tetradecadienyl acetate	98	4.4 ± 0.6 a	63	3.7 ± 0.2 a
Control	101	4.5 ± 0.6 a	63	3.8 ± 0.1 a
(*E*)-8-dodecenyl acetate	97	4.8 ± 0.8 a	62	3.4 ± 0.1 a

^1^ Number of sticky cards with data over the course of the season. ^2^ Letters represent multiple comparisons among semiochemical treatments within a year (χ^2^-test with Bonferroni correction).

## Data Availability

The data have been deposited and are accessible at Ag Data Commons at the following citation: Ruiz, Kaitlyn; Bruce, Alexander; Chérémond, Nervah C.; Stratton, Chase A.; Murrell, Ebony G.; Gillette, Samantha; Morrison, III William R.. Data from Field Trapping and Flight Capacity of *Eucosma giganteana* (Riley) (Lepidoptera: Tortricidae) in Response to Behaviorally Active Congeneric Semiochemicals in Novel Silflower Agroecosystems. Ag Data Commons. https://data.nal.usda.gov/dataset/data-field-trapping-and-flight-capacity-eucosma-giganteana-riley-lepidoptera-tortricidae-response-behaviorally-active-congeneric-semiochemicals-novel-silflower-agroecosystems, accessed on 21 March 2022.

## Supplementary Materials

The following supporting information can be downloaded at: https://www.mdpi.com/article/10.3390/insects13040350/s1, Supplemental Video S1: Flight of *E. giganteana* on automated flight mills in the Center for Grain and Animal Health Research in Manhattan, KS, USA.
